# Boosting the Activity of Melanoma-Targeting CAR-T Cells in the Presence of Citrate by the Application of Gluconate

**DOI:** 10.3390/pharmaceutics18050551

**Published:** 2026-04-30

**Authors:** Dennis Christoph Harrer, Sebastian Haferkamp, Wolfgang Herr, Maria Mycielska, Jan Dörrie, Niels Schaft, Hinrich Abken, Konstantin Drexler

**Affiliations:** 1 Department of Internal Medicine III—Hematology and Internal Oncology, University Hospital Regensburg, 93053 Regensburg, Germany; dennis.harrer@ukr.de (D.C.H.); wolfgang.herr@ukr.de (W.H.); 2 Bavarian Cancer Research Center (BZKF), 91054 Erlangen, Germany; jan.doerrie@uk-erlangen.de (J.D.); niels.schaft@uk-erlangen.de (N.S.); 3 CCC-WERA, 91054 Erlangen, Germany; sebastian.haferkamp@ukr.de; 4 Department of Dermatology, University Hospital Regensburg, 93053 Regensburg, Germany; 5 Department of Structural Biology, Institute of Biophysics and Physical Biochemistry, University of Regensburg, 93053 Regensburg, Germany; 6 Department Dermatology, Universitätsklinikum Erlangen, Friedrich-Alexander-Universität Erlangen-Nürnberg, 91054 Erlangen, Germany; 7 Comprehensive Cancer Center Erlangen European Metropolitan Area of Nuremberg (CCC ER-EMN), 91054 Erlangen, Germany; 8 Deutsches Zentrum Immuntherapie (DZI), 91054 Erlangen, Germany; 9 Leibniz Institute for Immunotherapy, Division Genetic Immunotherapy, Regensburg, and Chair Genetic Immunotherapy, University Regensburg, 93053 Regensburg, Germany; hinrich.abken@klinik.uni-regensburg.de

**Keywords:** CAR-T cell, adoptive T cell therapy, gluconate, citrate, melanoma, tumor microenvironment

## Abstract

**Background:** Chimeric antigen receptor (CAR) T cells achieve cure in the therapy of hematological malignancies. In solid tumors, however, CAR-T cells face an immunosuppressive tumor microenvironment (TME) which crucially impedes their cytotoxic capacities. Citrate accumulating in the TME is a crucial metabolite in mediating immune suppression and is consumed by cancer cells promoting growth of various tumors, including melanoma; blocking the citrate transporter pmCiC with gluconate abrogates citrate-mediated tumor growth. **Methods:** To bolster treatment of melanoma, we explored gluconate as adjuvant for CAR-T cell therapy. **Results:** First, gluconate did not impair CAR-T cell functional capacities with regard to cytotoxicity, cytokine secretion, and persistence in a “stress test” based on repetitive antigen stimulation with cognate cancer cells. The addition of gluconate antagonized the citrate-mediated enhanced proliferation of melanoma cells. As a consequence, the elimination of citrate-boosted melanoma cells by CSPG4-specific CAR-T cells was augmented in the presence of gluconate. **Conclusions:** Taken together, these data suggest that counteracting citrate-mediated enhanced tumor growth with gluconate may improve the cytotoxic activity of CAR-T cells against melanoma.

## 1. Introduction

Melanoma accounts for over 90% of skin cancer-related deaths [1]. Although prognosis is generally favorable in early stages of the disease, the management of metastatic disease still poses a substantial clinical challenge. With the development of immune checkpoint inhibitors, such as anti-programmed cell death protein 1 (anti-PD-1) and anti-cytotoxic T-lymphocyte-associated antigen 4 (anti-CTLA-4) antibodies, as well as targeted therapies involving BRAF and MEK inhibitors, the management of metastatic diseases substantially improved [1]. Despite these therapeutic advancements, approximately 55% of advanced melanoma patients show innate resistance to single-agent PD-1 inhibitors [2]. Even with combination therapy using CTLA-4 and PD-1 blockers, the innate resistance rate remains around 40% [3]. Among those who initially respond, about 25% develop resistance within the first two years [3]. Altogether, this means that roughly 70–80% of patients either fail to respond from the outset or relapse within two years, underscoring a significant unmet clinical need [2,3].

Adoptive therapy with tumor-derived, ex vivo expanded tumor infiltrating lymphocytes (TILs) or with T cells genetically engineered with a melanoma-specific T cell receptor (TCR) improved the prognosis of refractory melanoma patients [4]. A recent phase 3 multicenter trial demonstrated that TIL therapy conferred longer progression-free survival on patients with advanced melanoma than anti-CTLA-4 treatment, underscoring the power of adoptive T cell therapy in melanoma patients [5]. While TIL therapy depends on the successful isolation and amplification of T cells from the tumor tissue and the presence of a melanoma-specific TCR [4], chimeric antigen receptor (CAR) T cells are manufactured ex vivo from T cells from the circulating blood [5,6]. By means of the extracellular antigen-binding part, CAR-T cells engage the tumor-associated surface protein in an MHC-independent fashion, resulting in T cell activation and subsequent antigen-specific elimination of tumor cells [7]. Several CARs targeting melanoma cells, e.g., specific for chondroitin sulfate proteoglycan 4 (CSPG4) or tyrosinase-related protein 1 (TYRP1), have been elaborated in pre-clinical studies in recent years [8,9].

The success of CAR-redirected T cells for the treatment of solid tumors, however, is still limited. A crucial obstacle to the activity of CAR-T cells is the immunosuppressive tumor microenvironment (TME), mediated through myeloid-derived suppressor cells (MDSCs), regulatory T cells (Tregs), macrophages, and others [10]. In addition, soluble immunosuppressive metabolites impede the cytotoxicity of tumor-specific T cells [11] while sustaining the aggressiveness of melanoma cells and repressing the response to different therapeutics [12]. Citrate is one of the metabolites prevalent in the TME and plays a significant role in promoting cancer cell survival and tumor progression [13]. Citrate is taken up by cancer cells through a plasma membrane variant of the mitochondrial citrate transporter (pmCiC) [13,14,15]. The expression level of pmCiC correlates with tumor aggressiveness across several tumor entities [16,17]. Remarkably, gluconate is an irreversible inhibitor of pmCiC, capable of decreasing the uptake of citrate into cancer cells [15,18]. Correspondingly, the addition of gluconate to cancer cells reverted the growth-promoting effects of extracellular citrate.

Here, we aimed to boost the activity of melanoma-targeting CAR-T cells by the simultaneous application of gluconate. We provide the first evidence that gluconate does not impair canonical T cell functionality or CAR-T cell effector functions but preserves the cytotoxicity of CSPG4-specific CAR-T cells in the presence of citrate. We propose the use of gluconate to boost the cytotoxicity of CSPG4-CAR-T cells against melanoma.

## 2. Materials and Methods

### 2.1. Cells and Reagents

Peripheral blood mononuclear cells (PBMCs) were isolated by Lymphoprep^®^ centrifugation (Axis-Shield, Oslo, Norway) from healthy donors upon informed consent and approval by the institutional review board (21-2224-101 Regensburg). T cells were cultured in RPMI 1640 medium, 1% (*w*/*v*) GlutaMAX (Gibco, ThermoFisher, Waltham, MA, USA), 100 IU/mL penicillin, 100 µg/mL streptomycin (Pan-Biotech, Aidenbach, Germany), 2 mM HEPES (PAA, Palo Alto, CA, USA), and 10% (*v*/*v*) heat-inactivated fetal calf serum (FCS) (Pan-Biotech, Aidenbach, Germany). BxPC-3 cells (ATCC CRL-1420), 293T cells (ATCC CRL-3216 American Type Culture Collection, Manassas, VA, USA), A375 cells, A375M cells, and IGR39 cells were cultured in DMEM, 1% (*w*/*v*) GlutaMAX (Gibco, ThermoFisher), 100 IU/mL penicillin, 100 µg/mL streptomycin (Pan-Biotech), and 10% (*v*/*v*) heat-inactivated FCS (Sigma-Aldrich, St. Louis, MO, USA). Sodium-gluconate and citrate were purchased from Sigma (Sigma, St. Louis, MO, USA), dissolved in PBS, and stored in aliquots at −80 °C.

### 2.2. CAR-T Cell Generation

Cryopreserved blood lymphocytes were defrosted and activated with the anti-CD3 monoclonal antibody (mAb) OKT-3, the CD28 mAb 15E8, and IL-2 (1000 IU/mL). Recombinant IL-2 (200 IU/mL) was supplemented on days 2, 3, and 4. Retroviral transduction was performed as previously detailed [19]. In general, CAR-expressing T cells were isolated via magnetic activated cell sorting (MACS) (Miltenyi Biotec, Bergisch Gladbach, Germany). The CEA-specific CAR construct aCEA-28ζ and the aCSPG4-28ζ were previously published [20].

### 2.3. Flow Cytometry

Cells were stained with antibodies at 4 °C for 10 min. For intracellular staining, cells were processed using the “Transcription Factor Buffer Set” (BD Biosciences, Franklin Lakes, NJ, USA) for 20 min at 4 °C. The viability dye eFluor 780 (ThermoFisher, Waltham, MA, USA) was used for live/dead discrimination. Gates were set according to fluorescent-minus-one (FMO) controls. The goat F(ab’)2 anti-human IgG-PE antibody used to detect the CAR was purchased from SouthernBiotech, FITC-conjugated anti-CD8 (clone BW135/80), APC-conjugated anti-CD4 (clone VIT4), and PE-conjugated anti-CD25 (clone 4E3) were acquired from Miltenyi Biotec, and BV421-conjugated anti-CD8 (clone RPA-T8), BV421-conjugated anti-CD3 (clone OKT3), APC-conjugated anti-CD107a (clone DX22), and PE-conjugated anti-IL-2 (MQ1-17H12) were bought from Biolegend (San Diego, CA, USA). BV421-conjugated anti-CD137 (clone 4B4-1), PE-conjugated anti-human CSPG4 antibody (clone: 9.2.27), and BV421-conjugated anti-IFNγ (clone 4S.B3) were purchased from BD Biosciences (Franklin Lakes, NJ, USA). Immunofluorescence was determined using a BD FACSLyric (BD Biosciences). Data were analyzed using the FlowJo software version 10.7.1 Express 5 (BD Biosciences).

### 2.4. CD107a Degranulation Assay

Degranulation of T cells in response to the anti-CD3 monoclonal antibody OKT3 (2500 ng/mL) and the anti-CD28 monoclonal antibody 15E8 (5000 ng/mL) was measured using conventional CD107a staining. At the beginning of stimulation, either gluconate (g) was added at a concentration of 100 µM or PBS (w&o) was added, serving as a negative control. Monensin (BD Biosciences) and an APC-conjugated anti-CD107a antibody (Biolegend) were added at the beginning of co-culture. T cells were stained four hours later with the viability dye eFluor 780, a BV421-conjugated anti-CD8 antibody (BD), and an APC-conjugated anti-CD4 antibody (Miltenyi Biotec). The percentage of CD107a+ cells was analyzed via flow cytometry.

### 2.5. Cytokine Production

Intracellular cytokine staining for IFNγ and IL-2 in CD8^+^ and CD4^+^ T cells was performed following 6 h stimulation with the anti-CD3 monoclonal antibody OKT3 (2500 ng/mL) and the anti-CD28 monoclonal antibody 15E8 (5000 ng/mL) in the presence of Monensin (BD) and Brefeldin A (BD) used at concentrations as suggested by the manufacturer. At the beginning of stimulation, either gluconate (g) was added at a concentration of 100 µM or PBS (w&o) was added, serving as a negative control. In other experiments, target cells were seeded in 96-well round-bottom plates (1 × 10^5^ cells/well) overnight before adding CAR-T cells (1 × 10^5^ cells/well). After 48 h of co-incubation, IL-2 and IFNγ in culture supernatants were measured by ELISA. At the beginning of stimulation, either gluconate (g) was added at a concentration of 100 µM or PBS (w&o) was added, serving as a negative control.

### 2.6. Cytotoxicity Assay

CAR-T cells (0.125–10 × 10^4^ cells/well) were co-cultured for 24 h in 96-well round bottom plates with tumor cells (1 × 10^4^ cells/well) at decrementing effector-to-target ratios. In some experiments, 100 µM gluconate or 200 µM citrate were added at the beginning of the assay as indicated. The XTT-based colorimetric assay using the “Cell Proliferation Kit II” (Roche Diagnostics, Mannheim, Germany) was employed to determine specific cytotoxicity. The percentage of viable tumor cells in experimental wells was determined as follows: viability (%) = [OD (experimental wells − corresponding number of T cells)]/[OD (tumor cells without T cells − medium)] × 100. Cytotoxicity (%) was defined as 100 − viability (%).

### 2.7. Repetitive Stimulation Assay

GFP-expressing CEA^+^ BxPC-3 pancreatic carcinoma cells were plated at 10^5^ cells per well (12-well plate). Upon reaching 24 h, 10^5^ CAR-T cells were added. After three days (Round 1, R1), the cells were recovered from the wells and resuspended in 1 mL T cell medium. In total, 100 μL was employed for flow-cytometric cell counting (live GFP+ tumor cells and live CD3+/CAR+ CAR-T cells) using counting beads (“CountBright”, ThermoFisher). Then, 900 μL was transferred to a new 12-well plate with 10^5^ BxPC-3 cells for four days (round 2, R2). The procedure was reiterated for round 3 (R3). At the beginning of each round, either gluconate (g) was added at a concentration of 100 µM (CART + gluconate) or PBS was (CART + PBS). Further controls comprised PBS alone without CAR-T cells (PBS) and gluconate alone without CAR-T cells (gluconate).

### 2.8. Immunohistochemistry

As described before, Formalin-fixed paraffin-embedded tissue sections were deparaffinized in xylene (Merck, Darmstadt, Germany) and rehydrated through graded ethanol series (two washes each in 100%, 96%, and 70% ethanol) [21]. Endogenous peroxidase activity was quenched by incubation with 3% hydrogen peroxide for 10 min, followed by a rinse in distilled water. Antigen retrieval was performed by heating the sections in citrate buffer (pH 6; HIER, Zytomed/Biozol, Eching, Germany) at 90 °C for 20 min, after which samples were allowed to cool for 20 min. Blocking was carried out using the ZytoChem Plus HRP Kit (Rabbit/Mouse; Zytomed) for 10 min at room temperature.

Sections were then incubated overnight at 4 °C with a rabbit monoclonal anti-pmCiC antibody (D2P2F, Cell Signaling Technology; 1:200 for both patient and cell line samples) and a rabbit monoclonal anti-Ki67 antibody (Abcam, Cambridge, UK; 1:1000 for cell lines). Following washes with DPBS, slides were incubated with biotinylated anti-rabbit secondary antibodies (HRP060-RB) for 30 min, washed again in DPBS, and subsequently treated with streptavidin–HRP conjugate for 20 min, using components from the ZytoChem Plus HRP kit (Zytomed). Signal detection was performed with AEC+ High Sensitivity Substrate Chromogen (Dako/Agilent Technologies, Hamburg, Germany). Finally, sections were counterstained with hematoxylin (Carl Roth, Karlsruhe, Germany) and mounted using Aquatex (Merck).

### 2.9. Statistical Analysis

Statistical analysis was performed with GraphPad Prism, Version 9 (GraphPad Software, San Diego, CA, USA). *p* values were determined by Student’s *t*-test or two-way-ANOVA as indicated; ‘ns’ indicates not significant, * *p* ≤ 0.05, ** *p* ≤ 0.01, and *** *p* ≤ 0.001.

## 3. Results

### 3.1. Gluconate Does Not Impair Canonical T Cell Functionality

**Figure 1:**
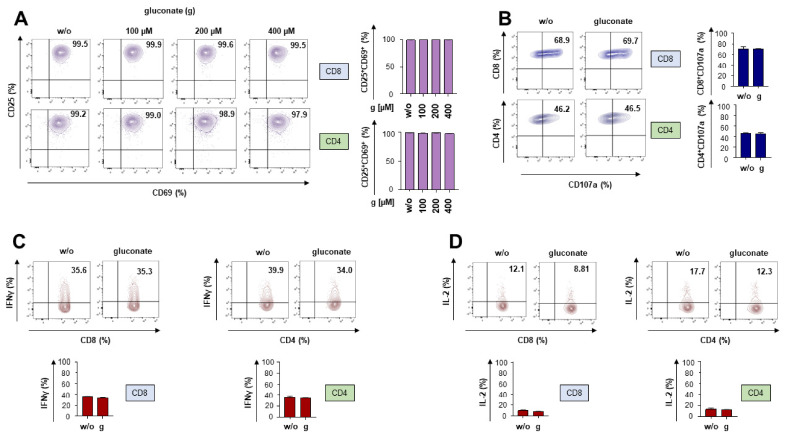
Gluconate does not impair canonical T cell functionality. (**A**) Upregulation of CD25 and CD69 on CD8^+^ and CD4^+^ T cells stimulated for 24 h with the anti-CD3 monoclonal antibody OKT3 (2500 ng/mL) and the anti-CD28 monoclonal antibody 15E8 (5000 ng/mL). At the beginning of stimulation, either gluconate (g) was added at the indicated concentrations or PBS (w/o) was, serving as a negative control. (**B**) Degranulation (staining for CD107a) of CD8^+^ and CD4^+^ T cells stimulated for 4 h with the anti-CD3 monoclonal antibody OKT3 (2500 ng/mL) and the anti-CD28 monoclonal antibody 15E8 (5000 ng/mL). At the beginning of stimulation, either gluconate (g) was added at a concentration of 100 µM or PBS (w/o) was, serving as a negative control. (**C**,**D**) Intracellular cytokine staining for IFNγ (**C**) and IL-2 (**D**) in CD8^+^ and CD4^+^ T cells stimulated for 6 h with the anti-CD3 monoclonal antibody OKT3 (2500 ng/mL) and the anti-CD28 monoclonal antibody 15E8 (5000 ng/mL). At the beginning of stimulation, either gluconate (g) was added at a concentration of 100 µM or PBS (w/o) was, serving as a negative control. (**A**–**D**) A dot plot of one representative donor out of three is shown (three biological replicates). Data represent means ± SEM of three T-cell donors (three biological replicates). Statistical significance was determined using Student’s *t*-test; all comparisons were not significant.

We aim to block the citrate transporter by gluconate during a CAR-T cell attack against melanoma cells. We first addressed whether adding gluconate impacts canonical T cell functionality. Peripheral blood T cells were expanded in the presence of IL-2 and subjected to TCR/CD28 stimulation through the anti-CD3 monoclonal antibody OKT3 and the anti-CD28 monoclonal antibody 15E8. Activation-induced T cell functionality was assayed in the presence of gluconate or PBS as a control. Gluconate at incrementing concentrations ranging from 100 to 400 µM was tested, with 100 µM being the established dose derived from previous work [21]. T cell activation as indicated by the upregulation of CD25 and CD69 was not affected by gluconate in CD8^+^ T cells or CD4^+^ T cells (Figure 1A). In all following experiments, the pre-established dose of 100 µM gluconate was used. Co-culture with gluconate did not exert any tangible impact on the upregulation of the activation marker CD137 or on the degranulation capacity of CD8^+^ and CD4^+^ T cells, as measured by staining for CD107a (Supplemental Figure S1 and Figure 1B). Finally, addition of gluconate did not impair the activation-induced release of the T cell cytokines IFNγ (Figure 1C) and IL-2 (Figure 1D). In aggregate, gluconate did not impair TCR/CD28 mediated T cell activation.

### 3.2. CAR-T Cells Maintain Functionality in the Presence of Gluconate

**Figure 2:**
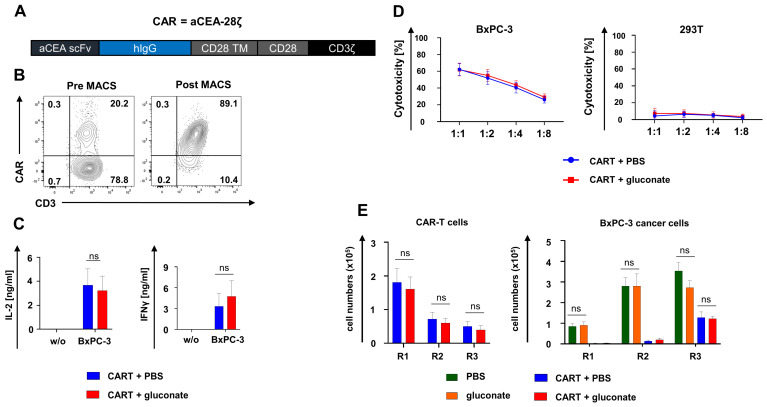
CAR-T cells maintain robust functionality in the presence of gluconate. (**A**) Schematic outline of the aCEA-28ζ CAR construct. (**B**) CAR-T cells were generated via retroviral transduction of bulk T cells obtained from healthy human donors. CAR expression on aCEA-28ζ CAR-T cells was determined prior to and after MACS. One representative donor out of three is depicted (three biological replicates). (**C**) CAR-triggered secretion of IL-2 and IFNγ from aCEA-28ζ CAR-T cells stimulated with BxPC-3 cells or left unstimulated (w/o) after 48 h, as measured by ELISA. At the beginning of stimulation, either gluconate was added at a concentration of 100 µM or PBS was, serving as a negative control. Data represent means ± SEM of three donors (three biological replicates), *p* values were calculated by two-way-ANOVA, ns indicates not significant. (**D**) Cytotoxicity of aCEA-28ζ CAR-T cells after a 24 h co-culture with BxPC-3 cells and 293T cells at the depicted effector to target cell ratios. At the beginning of co-culture, either gluconate was added at a concentration of 100 µM or PBS was, serving as a negative control. Data represent means ± SEM of three donors (three biological replicates), *p* values were calculated by two-way-ANOVA, ns indicates not significant. (**E**) In vitro “stress test”. Anti-CEA-28ζ CAR-T cells (1 × 10^5^ CAR-T cells) underwent three rounds (R1-R3) of incubation with GFP-labeled CEA^+^ BxPC-3 cells (1 × 10^5^ tumor cells at the start of each round). After each round, CAR-T cells (live CD3^+^ CAR cells) (left panel) and BxPC-3 cells (right panel) were enumerated by flow cytometry using counting beads. At the beginning of each round, either gluconate was added at a concentration of 100 µM (CART + gluconate) or PBS was (CART + PBS). Further controls comprised PBS alone without CAR-T cells (PBS) and gluconate alone without CAR-T cells (gluconate). Data represent means ± SEM of three donors (three biological replicates), *p* values were calculated by two-way-ANOVA, ns indicates not significant.

To characterize CAR-T cell functionality in the presence of gluconate, we employed a well-characterized second-generation CAR construct targeting carcinoembryonic antigen (CEA) referred to as aCEA-28ζ (Figure 2A). CAR-T cells were engineered by retroviral gene transfer and purified by MACS, achieving a homogenous population of CAR^+^ T cells for further experiments (Figure 2B). The release of IL-2 and IFNγ from aCEA-28ζ T cells upon co-incubation with CEA^+^ BxPC-3 pancreatic cancer cells did not significantly differ in the presence of 100 µM gluconate compared to the PBS control (Figure 2C). Furthermore, the addition of gluconate did not reduce the cytotoxicity of aCEA-28ζ T cells against CEA^+^ BxPC-3 cells or induce antigen-independent cytotoxicity against CEA^−^ 293T cells (Figure 2D). Finally, we evaluated the endurance of functional capacities in presence of gluconate upon repeated antigen stimulation of aCEA-28ζ T cells with CEA^+^ BxPC3 cells. At the beginning of the “stress test”, CAR-T cells proliferated before entering into a contraction phase without exhibiting significant quantitative differences, irrespective of gluconate supplementation (Figure 2E). In general, gluconate did not alter the cytolytic capacity of aCEA-28ζ CAR-T cells during the in vitro “stress test”. Gluconate alone did not impact the growth of CEA^+^ BxPC3 cells compared with the PBS control (Figure 2E). In summary, CAR-T cells maintained their robust functionality in the presence of gluconate in various in vitro assays.

### 3.3. Gluconate Antagonizes the Citrate-Mediated Enhanced Proliferation of Melanoma Cells

**Figure 3:**
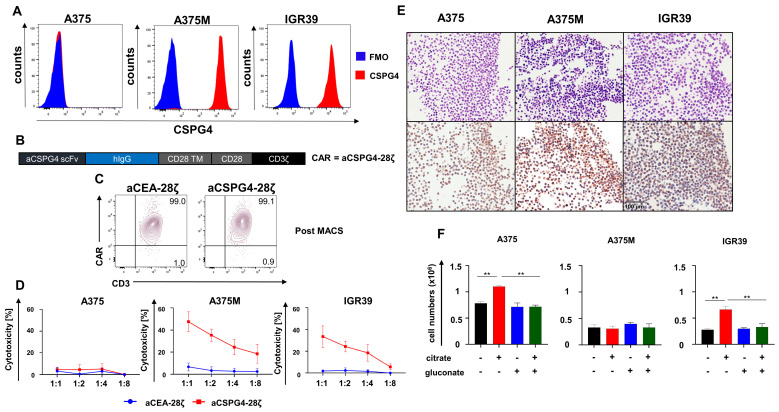
Gluconate antagonizes the citrate-mediated enhanced proliferation of melanoma cells. (**A**) Surface expression of CSPG4 on melanoma cells A375, A375M, and IGR39. One representative staining out of three independent experiments is shown (three biological replicates). (**B**) Schematic outline of the aCSPG4-28ζ CAR construct. (**C**) CAR-T cells were generated via retroviral transduction of bulk T cells followed by MACS isolation. CAR expression on aCSPG4-28ζ CAR-T cells was determined via flow cytometry. One representative donor out of three is shown (three biological replicates). (**D**) Cytotoxicity of aCSPG4-28ζ CAR-T cells relative to aCEA-28ζ CAR-T cells after a 24 h co-culture with A375 cells, A375M cells, and IGR39 cells at the depicted effector-to-target-cell ratios. Data represent means ± SEM of three donors (three biological replicates). (**E**) Expression of the citrate transporter pmCiC determined via immunohistochemistry in A375 cells, A375M cells, and IGR39 cells. Hematoxylin and eosin staining depicted in the upper panels and pmCiC staining in the lower panels. (**F**) Tumor cells (1 × 10^5^ cells) were seeded in 12-well plates and cultured for five days. Apart from PBS-treated controls, either 200 µM citrate, 100 µM gluconate, or the combination of 200 µM citrate and 100 µM gluconate was added. Tumor growth was determined after five days via conventional cell counting. Data represent means ± SEM of three runs (three biological replicates), *p* values were calculated by two-way-ANOVA, ** indicates *p* ≤ 0.01.

We addressed whether gluconate improves CAR-T cell activity against melanoma. Chondroitin sulfate proteoglycan 4 (CSPG4) has emerged as a prime target antigen on melanoma cells for CAR-T cell therapy. First, we confirmed the CSPG4 expression on melanoma cell lines by flow cytometry. While A375M and IGR39 melanoma cells displayed high CSPG4 levels, A375 did not show CSPG4 expression (Figure 3A). In order to redirect T cells towards CSPG4, we employed a second-generation CAR with specificity for CSPG4 called aCSPG4-28ζ (Figure 3B); aCEA-28ζ CAR-T cells served as control CAR-T cells of irrelevant specificity. Upon retroviral transduction, CAR-T cells were purified to obtain a homogenous population of CAR+ T cells (Figure 3C). T cells with aCSPG4-28ζ CAR antigen-specifically eliminated CSPG4^+^ A375M cells and CSPG4^+^ IGR39 cells while sparing CSPG4^−^ A375 cells. For comparison, aCEA-28ζ CAR-T cells did not exert any toxicity towards melanoma cells (Figure 3D).

Immunohistochemistry revealed that the used cell lines A375, A375M, and IGR39 express the citrate transporter pmCiC (Figure 3E). In order to interrogate the responsiveness of melanoma cells to citrate supplementation, the growth of A375, A375M, and IGR39 melanoma cell lines was recorded in the absence or presence of citrate (50 µM to 800 µM). While A375 and IGR39 showed augmented growth by addition of 100–800 µM citrate, A375M was not responsive to citrate supplementation (Supplemental Figure S2). The ensuing experiments were conducted with 200 µM citrate, resembling the concentration of citrate in the blood [22,23]. To specifically assess the reversibility of citrate-mediated enhanced proliferation of melanoma cells by gluconate, melanoma cells were incubated with 200 µM citrate, 100 µM gluconate, or both. The addition of citrate augmented the proliferation of A375 and IGR39 cells compared to PBS treatment, while no significant impact of citrate on the proliferation of A375M cells was recorded (Figure 3F). Remarkably, the simultaneous supply of gluconate nullified the citrate-mediated edge in the proliferation of both A375 cells and IGR39 cells (Figure 3F). Gluconate alone did not exert any effect on cell amplification. In sum, gluconate antagonizes the citrate-mediated enhanced proliferation of melanoma cells.

### 3.4. Gluconate Fosters the Cytotoxicity of Melanoma-Targeting CAR-T Cells in the Presence of Citrate

**Figure 4:**
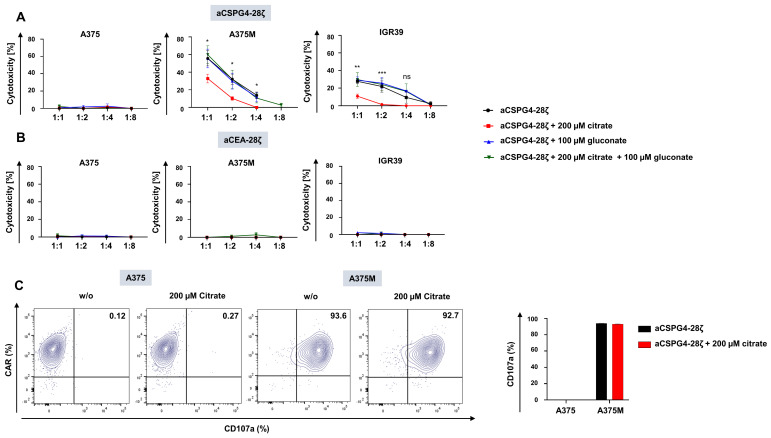
Gluconate fosters the cytotoxicity of melanoma-targeting CAR-T cells in the presence of citrate. (**A**,**B**) Cytotoxicity of aCSPG4-28ζ CAR-T cells (**A**) and aCEA-28ζ CAR-T cells (**B**) after a 24 h co-culture with A375 cells, A375M cells, and IGR39 cells at the depicted effector-to-target-cell ratios. At the beginning of co-culture, either PBS, 200 µM citrate, 100 µM gluconate, or the combination of 200 µM citrate and 100 µM gluconate was added. Data represent means ± SEM of three runs (three biological replicates), *p* values were calculated by two-way-ANOVA, * indicates *p* ≤ 0.05, ** *p* ≤ 0.01, and *** *p* ≤ 0.001, ns indicates not significant. (**C**) Degranulation (staining for CD107a) of CAR+ T cells cultured for 4 h with A375 and A375M cells. At the beginning of stimulation, either citrate was added a concentration of 200 µM or PBS was (w/o), serving as a negative control. A dot plot of one representative donor out of three is shown (three biological replicates). Data represent means ± SEM of three T-cell donors (three biological replicates). Statistical significance was determined using two-way-ANOVA; the comparison was not significant.

We assayed the cytotoxicity of aCSPG4-28ζ CAR-T cells against melanoma cells in the presence of citrate and gluconate. Consistent with previous results, CSPG4- A375 cells were not killed by aCSPG4-28ζ CAR-T cells (Figure 4A). By contrast, A375M and IGR39 were efficiently lysed by CSPG4-specific CAR-T cells (Figure 4A). Notably, the presence of 200 µM citrate significantly reduced the elimination of A375M cells and IGR39 cells by aCSPG4-28ζ CAR T-cells, indicating a protective effect even in the A375M cells, which did not show a citrate-induced growth advantage. Importantly, the addition of 100 µM gluconate restored the cytolytic capacity of aCSPG4-28ζ CAR-T cells in the presence of citrate (Figure 4A), providing evidence for the combined use of CAR-T cells and gluconate against melanoma. Supplemented gluconate alone did not augment the cytotoxicity of aCSPG4-28ζ CAR-T cells above baseline (Figure 4A). Of note, killing assays were performed in R10 medium, which does not contain citrate at biologically relevant concentrations. Control aCEA-28ζ CAR-T cells did not evince cytotoxicity to melanoma cells, irrespective of citrate or gluconate supplementation (Figure 4B). To examine whether citrate directly affects CAR-T cell functionality, they were co-incubated with A375 and A375M cells with or without the addition of 200 µM citrate, and degranulation was measured via CD107a staining. In accordance with the XTT assay, CSPG4-negative A375 cells did not trigger CAR-T-cell degranulation, while CSPG4-positive A375M cells induced pronounced CAR-T cell degranulation (Figure 4C). Importantly, CAR-T cell degranulation was not affected by citrate, pointing to a T-cell extrinsic resistance mechanism to account for the decreased CAR T cell cytotoxicity towards melanoma cells in the presence of citrate. In aggregate, gluconate preserved the cytotoxicity of CSPG4-specific CAR-T cells against melanoma cells in the presence of citrate.

## 4. Discussion

Gluconate-mediated blockade counteracts the tumor-promoting effects of citrate on cancer cell amplification. To strengthen the power of CAR-T cell therapy in melanoma, we evaluated the co-administration of gluconate as a potential avenue to boost the cytolytic capacity of melanoma-targeting CSPG4-specific CAR-T cells. While gluconate did not exert any detrimental effects on CAR-T cell functionality, the citrate-enhanced proliferation of melanoma cells was significantly inhibited and the elimination of melanoma cells by CSPG4-specific CAR-T cells in the presence of citrate was significantly improved. As the impact of gluconate on CAR T-cell functionality has not previously been investigated, we adopted a sequential experimental approach. First, we confirmed that gluconate does not impair the functionality of untransduced T cells. Second, we employed the well-established CEA-CAR platform as a model system to demonstrate that gluconate does not interfere with CAR T-cell activity in principle. While CEA is predominantly expressed in gastrointestinal malignancies and only rarely detected in melanoma [24,25], this system allowed us to establish, in a paradigmatic manner, that gluconate does not compromise CAR-mediated cytotoxicity. Finally, we evaluated CAR T cells targeting the melanoma-associated antigen CSPG4, which is expressed on approximately 90% of melanoma lesions [26] and has been validated in several preclinical melanoma studies [25,27,28]. Using this melanoma-relevant setting, we demonstrate that gluconate can abrogate the citrate-induced growth advantage of melanoma cells and enhance CAR T-cell cytotoxic activity against melanoma.

Generally, the membrane impermeant anion gluconate is widely used in medicine as an inert carrier of cations, such as Zn^2+^, Ca^2+^, and Cu^2+^, for electrolyte substitution therapy [29]. Moreover, gluconic acid is a natural ingredient of fruits, honey, and wine which can be safely incorporated without any side effects [29]. In addition, stibogluconate, a derivative of gluconate, is part of the treatment regimen for leishmaniasis [30]. Clinical trials involving the use of gluconate in cancer patients encompass the co-administration of Zn^2+^ gluconate with chemotherapy against acute lymphoblastic leukemia [31], co-supplementation of Zn^2+^ gluconate with cytokine therapy against squamous cell carcinoma [32], and co-infusion of Ca^2+^ gluconate to mitigate oxaliplatin-induced polyneuropathy [33]. Finally, Zn^2+^ gluconate was successfully partnered with Disulfiram in a patient with liver metastases owing to ocular melanoma achieving a significant survival benefit [34]. Collectively, these observations indicate that gluconate is a well-tolerated compound with extensive clinical experience and minimal serious side effects. From a pharmacokinetic perspective, gluconate is a small, highly water-soluble molecule that is largely cleared through renal excretion and undergoes minimal metabolic conversion in humans. Owing to its low molecular weight, hydrophilicity, and similarity to glucose, gluconate is also expected to distribute readily from the circulation into interstitial compartments, suggesting that pharmacologically relevant concentrations may diffuse into the tumor microenvironment. Against the backdrop that gluconate-containing formulations such as Ca^2+^ gluconate are routinely administered intravenously at gram-scale doses [35,36], we presume that substantial systemic exposure can be achieved without major toxicity. Hence, the required concentration of 100 µM is presumed to be feasible and tolerable both in the serum and the TME.

A primary source for extracellular citrate in the TME is cancer-associated fibroblasts, which produce citrate via reverse carboxylation from glutamine and release citrate via the pmCiC [18]. Extracellular citrate is an immune-cell-suppressive metabolite in the TME of solid tumors and a stimulator for cancer cell growth upon uptake via the pmCiC, as demonstrated for prostate [37], gastric [15], pancreatic [15], hepatic [15], and merkel cell carcinoma [21]. The virtually omnipresent expression of pmCiC by cancer cells and stromal cells suggests citrate uptake as a mechanism to cope with metabolic needs during neoplastic progression. For the addition of citrate in the in vitro assays, we used the concentration of 200 µM citrate as it is the approximate concentration of citrate in the blood [29] and is used in other in vitro studies [18,21]. The supply of extracellular citrate was shown to aid in metastasis by spawning invasive phenotypes in cancer cells to facilitate metastatic spread [15]. Hence, the decrease in extracellular citrate uptake via pmCiC blockade could exert a two-pronged attack on cancer cells by curbing tumor cell metabolism on the one hand and decreasing metastatic potential on the other hand. With A375 and IGR39 melanoma cells, we verify the sensitivity of melanoma cells to extracellular citrate and gluconate in vitro.

In this context, it is of particular interest that the cytotoxicity of CSPG4-specific CAR-T cells was preserved by adding gluconate. Mechanistically, we hypothesize that citrate uptake enhances the proliferative capacity of tumor cells, thereby exhausting the cytolytic capacity of CAR T cells by shifting toward lower effector-to-target ratios. With regard to the A375M cell line, we observed that these cells did not exhibit citrate-induced proliferation under standard culture conditions. Nevertheless, A375M cells express the plasma membrane citrate transporter pmCiC, indicating that the molecular machinery for extracellular citrate uptake is present. This suggests that under basal in vitro conditions, A375M cells may not strongly depend on extracellular citrate. However, under conditions of cellular stress, such as immune pressure exerted by CAR-T cells, tumor cells may undergo metabolic adaptation and increase their reliance on alternative extracellular nutrient sources [15,38], including citrate via pmCiC. Such stress-induced metabolic rewiring could render citrate uptake functionally relevant and contribute to reduced CAR-T cytotoxicity by citrate-induced enhanced proliferation, resulting in a shift toward lower effector-to-target ratios. In this context, the inhibition of citrate uptake by gluconate may restore CAR-T activity. All in all, gluconate maintains CAR-T cell cytotoxicity in the presence of citrate. In the absence of citrate, CAR-T cells in the presence of gluconate did not show an enhanced killing capacity relative to CAR-T cells alone. This finding corroborates the hypothesis that gluconate merely counteracts the citrate-mediated resistance to CAR-T cell-mediated killing without unfolding cytotoxic capacity towards tumor cells or augmenting CAR-T cytotoxicity per se. Clinically, co-administering gluconate with CAR-T cells has the benefit of the absence of functional constraints on CAR-T cell activity, along with a virtual lack of severe side effects.

A key limitation of the present study is that all experiments were performed in vitro, and therefore the findings do not capture the full complexity of the tumor microenvironment in vivo. In particular, extracellular citrate levels within the TME are influenced by tumor progression, stromal development, and metabolic interactions between tumor and host cells. Consequently, appropriate in vivo validation of the mechanism investigated here would require tumor models capable of recapitulating these metabolic features. For example, syngeneic mouse models such as the B16 melanoma model with sufficiently established tumor growth would likely be necessary to allow stromal development and accumulation of relevant extracellular citrate concentrations [39]. Short-term tumor growth upon xenogeneic tumor cell implantation will not address the biology as studied here in vitro. Therefore, the present study should be considered a proof-of-concept investigation that provides a rationale for future in vivo and mechanistic studies evaluating the potential of targeting citrate transport to modulate CAR T-cell-mediated tumor cell killing.

## 5. Conclusions

In aggregate, we revealed the application of gluconate along with CAR-T cells to augment anti-melanoma cell cytotoxicity. Mechanistically, gluconate blocks the citrate carrier pmCiC to decrease the uptake of extracellular citrate into melanoma cells, which is assumed to desensitize melanoma cells against apoptosis induction upon a CAR-T cell attack. The addition of gluconate antagonized the effects of citrate on tumor cells, thereby preserving CAR-T cell cytotoxicity in the presence of citrate. Being void of known severe side effects and being widely used as a cation carrier, gluconate can be clinically applied in standard CAR-T cell therapy in order to augment cytotoxic activity against solid cancer.

## Data Availability

Original datasets are available from the corresponding author on reasonable request.

## Supplementary Materials

The following supporting information can be downloaded at: https://www.mdpi.com/article/10.3390/pharmaceutics18050551/s1, Figure S1: Gluconate does not impair canonical T cell activation; Figure S2: Citrate augments the proliferation of melanoma cells.

## Abbreviations

The following abbreviations are used in this manuscript:

BRAF	B-Raf proto-oncogene
CAR	Chimeric antigen receptor
CD	Cluster of differentiation
CEA	Carcinoembryonic antigen
CSPG4	Chondroitin Sulfate Proteoglycan 4
CTLA-4	Cytotoxic T-Lymphocyte-Associated Protein 4
FCS	Fetal calf serum
FMO	Fluorescent-minus-one
G	Gluconate
IL	Interleukin
Mab	Monoclonal antibody
MACS	Magnetic-activated cell sorting
MDSCs	Myeloid-derived suppressor cells
MEK	Mitogen-activated protein kinase
MHC	Major histocompatibility complex
PBMCs	Peripheral blood mononuclear cells
PBS	Phosphate-buffered saline
PD-1	Programmed death 1
pmCiC	Citrate transporter on plasma membrane
ROS	Reactive oxygen species
TCR	T cell receptor
TIL	Tumor-Infiltrating Lymphocyte
TME	Tumor microenvironment
Tregs	T regulatory cells
TYRP1	Tyrosinase-related protein 1
w/o	Without

## References

[1] Connor C., Carr Q.L., Sweazy A., McMasters K., Hao H. (2025). Clinical Approaches for the Management of Skin Cancer: A Review of Current Progress in Diagnosis, Treatment, and Prognosis for Patients with Melanoma. Cancers.

[2] Wang T., Ma W., Zou Z., Zhong J., Lin X., Liu W., Sun W., Hu T., Xu Y., Chen Y. (2025). PD-1 Blockade Treatment in Melanoma: Mechanism of Response and Tumor-Intrinsic Resistance. Cancer Sci..

[3] Vukadin S., Khaznadar F., Kizivat T., Vcev A., Smolic M. (2021). Molecular Mechanisms of Resistance to Immune Checkpoint Inhibitors in Melanoma Treatment: An Update. Biomedicines.

[4] Guedan S., Ruella M., June C.H. (2019). Emerging Cellular Therapies for Cancer. Annu. Rev. Immunol..

[5] Rohaan M.W., Borch T.H., van den Berg J.H., Met Ö., Kessels R., Geukes Foppen M.H., Stoltenborg Granhøj J., Nuijen B., Nijenhuis C., Jedema I. (2022). Tumor-Infiltrating Lymphocyte Therapy or Ipilimumab in Advanced Melanoma. N. Engl. J. Med..

[6] June C.H., Sadelain M. (2018). Chimeric Antigen Receptor Therapy. N. Engl. J. Med..

[7] Majzner R.G., Mackall C.L. (2018). Tumor Antigen Escape from CAR T-cell Therapy. Cancer Discov..

[8] Jilani S., Saco J.D., Mugarza E., Pujol-Morcillo A., Chokry J., Ng C., Abril-Rodriguez G., Berger-Manerio D., Pant A., Hu J. (2024). CAR-T Cell Therapy Targeting Surface Expression of TYRP1 to Treat Cutaneous and Rare Melanoma Subtypes. Nat. Commun..

[9] Harrer D.C., Dörrie J., Schaft N. (2019). CSPG4 as Target for CAR-T-Cell Therapy of Various Tumor Entities-Merits and Challenges. Int. J. Mol. Sci..

[10] de Visser K.E., Joyce J.A. (2023). The Evolving Tumor Microenvironment: From Cancer Initiation to Metastatic Outgrowth. Cancer Cell.

[11] Metallo C.M., Gameiro P.A., Bell E.L., Mattaini K.R., Yang J., Hiller K., Jewell C.M., Johnson Z.R., Irvine D.J., Guarente L. (2011). Reductive Glutamine Metabolism by IDH1 Mediates Lipogenesis under Hypoxia. Nature.

[12] Tian J., Quek C. (2024). Understanding the Tumor Microenvironment in Melanoma Patients with In-Transit Metastases and Its Impacts on Immune Checkpoint Immunotherapy Responses. Int. J. Mol. Sci..

[13] Petillo A., Abruzzese V., Koshal P., Ostuni A., Bisaccia F. (2020). Extracellular Citrate Is a Trojan Horse for Cancer Cells. Front. Mol. Biosci..

[14] Mazurek M.P., Prasad P.D., Gopal E., Fraser S.P., Bolt L., Rizaner N., Palmer C.P., Foster C.S., Palmieri F., Ganapathy V. (2010). Molecular Origin of Plasma Membrane Citrate Transporter in Human Prostate Epithelial Cells. EMBO Rep..

[15] Mycielska M.E., Dettmer K., Rümmele P., Schmidt K., Prehn C., Milenkovic V.M., Jagla W., Madej G.M., Lantow M., Schladt M. (2018). Extracellular Citrate Affects Critical Elements of Cancer Cell Metabolism and Supports Cancer Development In Vivo. Cancer Res..

[16] Schwertner B., Dahdal G., Jagla W., Grossmann L., Drexler K., Krahn M.P., Evert K., Berneburg M., Haferkamp S., Ziegler C. (2024). Expression of the Plasma Membrane Citrate Carrier (pmCiC) in Human Cancerous Tissues-Correlation with Tumour Aggressiveness. Front. Cell Dev. Biol..

[17] Drexler K., Schwertner B., Zenderowski V., Schreieder L., Harrer D.C., Berneburg M., Geissler E., Mycielska M., Haferkamp S. (2026). The Influence of Extracellular Citrate in Physiological Concentration on the Proliferation of Malignant Melanoma. J. Cell. Mol. Med..

[18] Drexler K., Schmidt K.M., Jordan K., Federlin M., Milenkovic V.M., Liebisch G., Artati A., Schmidl C., Madej G., Tokarz J. (2021). Cancer-Associated Cells Release Citrate to Support Tumour Metastatic Progression. Life Sci. Alliance.

[19] Harrer D.C., Schenkel C., Berking C., Herr W., Abken H., Dörrie J., Schaft N. (2022). Decitabine-Mediated Upregulation of CSPG4 in Ovarian Carcinoma Cells Enables Targeting by CSPG4-Specific CAR-T Cells. Cancers.

[20] Harrer D.C., Schlierkamp-Voosen T., Barden M., Pan H., Xydia M., Herr W., Dörrie J., Schaft N., Abken H. (2025). Chimeric Antigen Receptor (CAR) T Cells Releasing Soluble SLAMF6 Isoform 2 Gain Superior Anti-Cancer Cell Functionality in an Auto-Stimulatory Fashion. Cells.

[21] Drexler K., Schwertner B., Haerteis S., Aung T., Berneburg M., Geissler E.K., Mycielska M.E., Haferkamp S. (2022). The Role of Citrate Homeostasis in Merkel Cell Carcinoma Pathogenesis. Cancers.

[22] Costello L.C., Franklin R.B. (2016). Plasma Citrate Homeostasis: How It Is Regulated; And Its Physiological and Clinical Implications. An Important, But Neglected, Relationship in Medicine. HSOA J. Hum. Endocrinol..

[23] Kumar A., Cordes T., Thalacker-Mercer A.E., Pajor A.M., Murphy A.N., Metallo C.M. (2021). NaCT/SLC13A5 Facilitates Citrate Import and Metabolism under Nutrient-Limited Conditions. Cell Rep..

[24] Abken H. (2025). CAR T Cell Therapies in Gastrointestinal Cancers: Current Clinical Trials and Strategies to Overcome Challenges. Nat. Rev. Gastroenterol. Hepatol..

[25] Harrer D.C., Simon B., Fujii S.-I., Shimizu K., Uslu U., Schuler G., Gerer K.F., Hoyer S., Dörrie J., Schaft N. (2017). RNA-Transfection of γ/δ T Cells with a Chimeric Antigen Receptor or an α/β T-cell Receptor: A Safer Alternative To Genetically Engineered α/β T Cells for the Immunotherapy of Melanoma. BMC Cancer.

[26] Campoli M.R., Chang C.-C., Kageshita T., Wang X., McCarthy J.B., Ferrone S. (2004). Human High Molecular Weight-Melanoma-Associated Antigen (HMW-MAA): A Melanoma Cell Surface Chondroitin Sulfate Proteoglycan (MSCP) with Biological and Clinical Significance. Crit. Rev. Immunol..

[27] Krug C., Birkholz K., Paulus A., Schwenkert M., Schmidt P., Hoffmann N., Hombach A., Fey G., Abken H., Schuler G. (2015). Stability and Activity of MCSP-Specific Chimeric Antigen Receptors (CARs) Depend on the scFv Antigen-Binding Domain and the Protein Backbone. Cancer Immunol. Immunother..

[28] Wiesinger M., März J., Kummer M., Schuler G., Dörrie J., Schuler-Thurner B., Schaft N. (2019). Clinical-Scale Production of CAR-T Cells for the Treatment of Melanoma Patients by mRNA Transfection of a CSPG4-Specific CAR under Full GMP Compliance. Cancers.

[29] Mycielska M.E., Mohr M.T.J., Schmidt K., Drexler K., Rümmele P., Haferkamp S., Schlitt H.J., Gaumann A., Adamski J., Geissler E.K. (2019). Potential Use of Gluconate in Cancer Therapy. Front. Oncol..

[30] Alves F., Bilbe G., Blesson S., Goyal V., Monnerat S., Mowbray C., Muthoni Ouattara G., Pécoul B., Rijal S., Rode J. (2018). Recent Development of Visceral Leishmaniasis Treatments: Successes, Pitfalls, and Perspectives. Clin. Microbiol. Rev..

[31] Eby G.A. (2005). Treatment of Acute Lymphocytic Leukemia Using Zinc Adjuvant with Chemotherapy and Radiation—A Case History and Hypothesis. Med. Hypotheses.

[32] Freeman S.M., Franco J.L.B., Kenady D.E., Baltzer L., Roth Z., Brandwein H.J., Hadden J.W. (2011). A Phase 1 Safety Study of an IRX-2 Regimen in Patients with Squamous Cell Carcinoma of the Head and Neck. Am. J. Clin. Oncol..

[33] Gamelin L., Boisdron-Celle M., Delva R., Guérin-Meyer V., Ifrah N., Morel A., Gamelin E. (2004). Prevention of Oxaliplatin-Related Neurotoxicity by Calcium and Magnesium Infusions: A Retrospective Study of 161 Patients Receiving Oxaliplatin Combined with 5-Fluorouracil and Leucovorin for Advanced Colorectal Cancer. Clin. Cancer Res. Off. J. Am. Assoc. Cancer Res..

[34] Brar S.S., Grigg C., Wilson K.S., Holder W.D., Dreau D., Austin C., Foster M., Ghio A.J., Whorton A.R., Stowell G.W. (2004). Disulfiram Inhibits Activating Transcription Factor/Cyclic AMP-Responsive Element Binding Protein and Human Melanoma Growth in a Metal-Dependent Manner in Vitro, in Mice and in a Patient with Metastatic Disease. Mol. Cancer Ther..

[35] Zhao Y., Linden J., Welch L., St Pierre P., Graves M., Garrity D., Ducharme P., Bailey J.A., Greene M., Vauthrin M. (2018). Prophylactic Infusion of Calcium Gluconate to Prevent a Symptomatic Fall in Plasma Ionized Calcium during Therapeutic Plasma Exchange: A Comparison of Two Methods. J. Clin. Apher..

[36] Zhao Y., Garrity D., Graves M., Linden J., St Pierre P., Ducharme P., Greene M., Vauthrin M., Weinstein R. (2019). Optimization of Infusional Calcium Gluconate for Prevention of Hypocalcemic Reactions during Therapeutic Plasma Exchange. J. Clin. Apher..

[37] Mycielska M.E., Palmer C.P., Brackenbury W.J., Djamgoz M.B.A. (2005). Expression of Na^+^-Dependent Citrate Transport in a Strongly Metastatic Human Prostate Cancer PC-3M Cell Line: Regulation by Voltage-Gated Na^+^ Channel Activity. J. Physiol..

[38] Parkinson E.K., Adamski J., Zahn G., Gaumann A., Flores-Borja F., Ziegler C., Mycielska M.E. (2021). Extracellular Citrate and Metabolic Adaptations of Cancer Cells. Cancer Metastasis Rev..

[39] Baldwin J.G., Heuser-Loy C., Saha T., Schelker R.C., Slavkovic-Lukic D., Strieder N., Hernandez-Lopez I., Rana N., Barden M., Mastrogiovanni F. (2024). Intercellular Nanotube-Mediated Mitochondrial Transfer Enhances T Cell Metabolic Fitness and Antitumor Efficacy. Cell.

