# Supplementary figures and images for: Boosting the Activity of Melanoma-Targeting CAR-T Cells in the Presence of Citrate by the Application of Gluconate
Source: Pharmaceutics. 2026 Apr 30;18(5):551. doi: 10.3390/pharmaceutics18050551 (PMC13211162; doi:10.3390/pharmaceutics18050551)

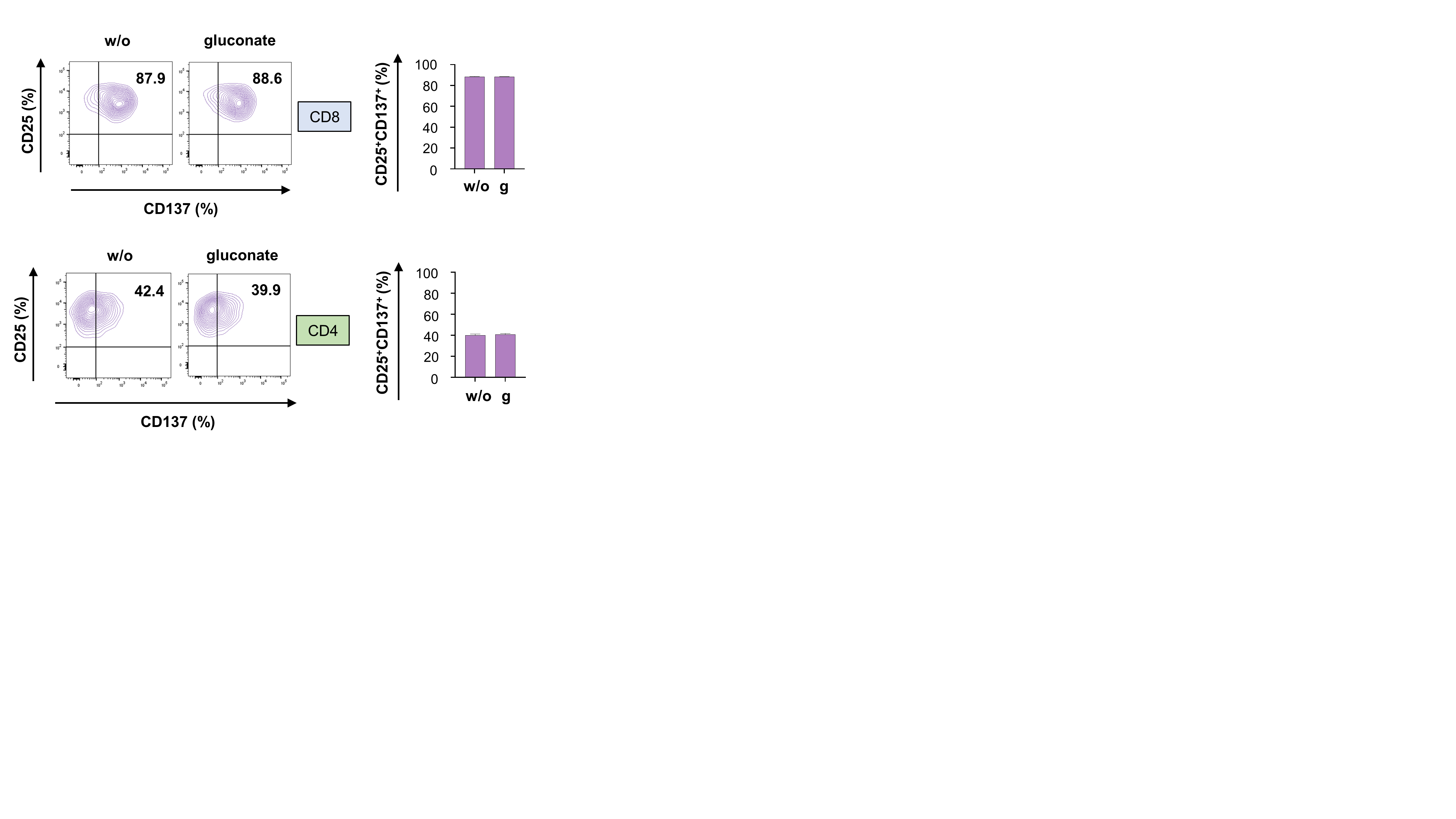

Supplement: Supplementary file 1 [file pharmaceutics-18-00551-s001.zip › Supplemental Figure S1.tif]

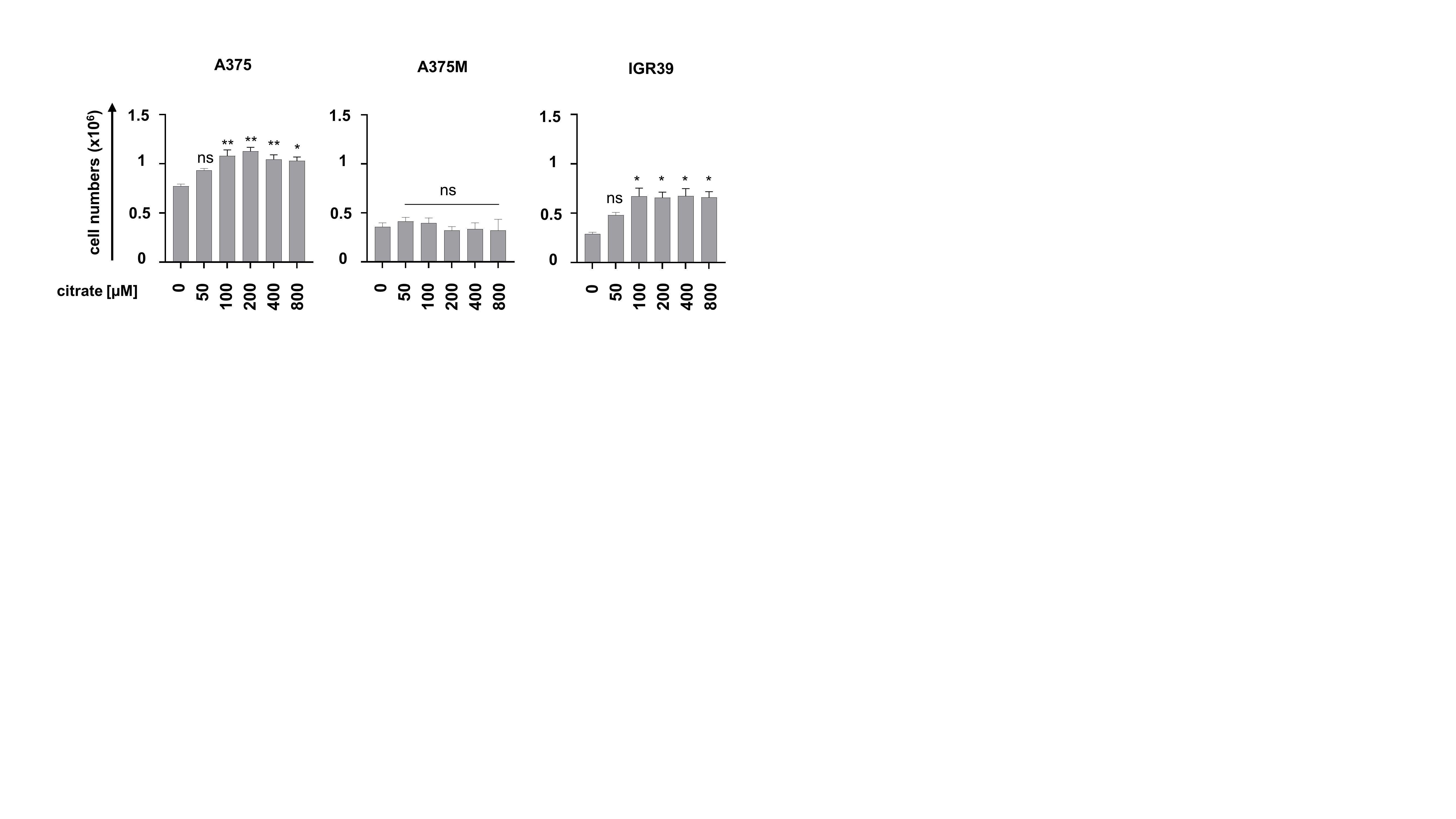

Supplement: Supplementary file 1 [file pharmaceutics-18-00551-s001.zip › Supplemental Figure S2.tif]
